# Impact of Von Willebrand Factor on Bacterial Pathogenesis

**DOI:** 10.3389/fmed.2020.00543

**Published:** 2020-09-03

**Authors:** Michael Steinert, Isabell Ramming, Simone Bergmann

**Affiliations:** ^1^Institut für Mikrobiologie, Technische Universität Braunschweig, Braunschweig, Germany; ^2^Department of Infection Biology, Helmholtz Center for Infection Diseases, Braunschweig, Germany

**Keywords:** von Willebrand, *Staphylococcus areus*, *Streptococcus pneumoniae*, microfluidic, sepsis

## Abstract

Von Willebrand factor (VWF) is a mechano-sensitive protein with crucial functions in normal hemostasis, which are strongly dependant on the shear-stress mediated defolding and multimerization of VWF in the blood stream. Apart from bleeding disorders, higher plasma levels of VWF are often associated with a higher risk of cardiovascular diseases. Herein, the disease symptoms are attributed to the inflammatory response of the activated endothelium and share high similarities to the reaction of the host vasculature to systemic infections caused by pathogenic bacteria such as *Staphylococcus aureus* and *Streptococcus pneumoniae*. The bacteria recruit circulating VWF, and by binding to immobilized VWF on activated endothelial cells in blood flow, they interfere with the physiological functions of VWF, including platelet recruitment and coagulation. Several bacterial VWF binding proteins have been identified and further characterized by biochemical analyses. Moreover, the development of a combination of sophisticated cell culture systems simulating shear stress levels of the blood flow with microscopic visualization also provided valuable insights into the interaction mechanism between bacteria and VWF-strings. *In vivo* studies using mouse models of bacterial infection and zebrafish larvae provided evidence that the interaction between bacteria and VWF promotes bacterial attachment, coagulation, and thrombus formation, and thereby contributes to the pathophysiology of severe infectious diseases such as infective endocarditis and bacterial sepsis. This mini-review summarizes the current knowledge of the interaction between bacteria and the mechano-responsive VWF, and corresponding pathophysiological disease symptoms.

## Introduction

Vascular hemostasis is a live-saving mechanism, which balances coagulation, thrombogenesis, and fibrinolysis in response to vascular injuries and inflammatory processes. Key element of the hemostasis are the Weibel Palade bodies (WPBs), which represent defense vesicles, constitutively produced by the endothelium of the vessel walls. The vesicles are filled with vasoactive substances, immune defense modulators, and proteins involved in coagulation (1, 2). In addition to megakaryocytes, endothelial WPBs are the main source of Von Willebrand factor (VWF). This glycoprotein mediates platelet activation, anchorage of thrombocytes to the subendothelial collagen, and induction of plasma haemostasis via factor VIII (3, 4). Moreover, VWF promotes cell migration in angiogenesis via interaction with different cell surface receptors and induction of signaling pathways (5). The high importance of VWF for balanced hemostasis is conveyed by the appearance of bleeding disorders such as the von Willebrand disease caused by an inherited quantitative or functional VWF deficiency (3).

VWF constantly circulates in the bloodstream at concentrations between 8 and 14.0 μg/mL (3, 6). But, vasoactive hormones such as epinephrine and vasopressin as well as the plasma proteins thrombin, histamine, and numerous other mediators of inflammation and/or thrombosis induce the release of VWF in response to vascular injury or inflammatory stimuli. The released VWF increases the plasma levels of this protein, and some proportion of VWF is temporarily retained on the cell surface and binds to collagen of the exposed subendothelial matrix (7, 8). This subendothelial immobilization is also significantly strengthened by the endothelial glycocalyx in a heparanase-sensitive manner (9). VWF is a mechano-sensitive protein, which responds to shear stress-mediated forces by conformational changes.

Shear stress is defined as the force exerted by the blood flow on blood vessel walls. This stress generates a response in the vascular wall, characterized by release of endothelial mediators, which in turn stimulate structural remodeling through activation of gene expression and protein synthesis (10). The shear stress-derived conformational changes of VWF are crucial for the biological function of VWF in hemostasis. Upon exposition to the shear forces in the bloodstream, the immobilized VWF unfolds to large protein strings, thereby exposing further functionally important binding sites (7, 11, 12). In particular, the defolded A1 binding site mediates adhesion of platelets and recruits them via binding to the platelet glycoprotein GP?bα (11, 13, 14). This VWF-platelet interaction finally results in a factor VIII-induced fibrin-incorporation and in stabilization of generated thrombi.

Elevated VWF-levels are directly associated with cardiovascular diseases (CD) of high-risk groups such as the elderly and diabetes patients (15). Alongside with tissue plasminogen activator (t-PA), and D-dimer of fibrinogen, VWF is characterized as one out of three biomarkers directly associated with atherosclerotic lesions and coronary heart disease (16, 17). This unveils the thrombus-generating activity of elevated VWF-concentrations as one of the dominant causative factors for coronary heart disease (18).

In addition to the role of VWF in CD, VWF serves as a ligand binding site for bacteria, which cause live-threatening local and systemic infection diseases, such as *Staphylococcus aureus* and *Streptococcus pneumoniae* (19, 20). *S. aureus* is a human pathogenic bacterium causing, among others, infective endocarditis and heart valve prosthetic infection (21, 22). In this respect, shear-force-mediated adhesion of staphylococci to VWF is directly associated with coagulation and typical disease symptoms (23, 24). Similarly, *S. pneumoniae*, a commensal colonizing the upper respiratory epithelium and a major cause of community-acquired pneumoniae in elderly and immunocompromised patients (25, 26), has also been recurringly isolated from heart valve endocardium of patients suffering from subacute endocarditis (27, 28). Furthermore, an increasing amount of clinical case studies report that up to one-third of patients suffer from major adverse cardiac effects (MACE) and vascular impairments within months and even years after recovering from severe pneumococcal infections such as pneumoniae and septicemiae (29–31). The observation of similarities between the association of CD with VWF-release, and symptoms induced by bacterial infections initiated an increasing need to develop infection models and sophisticated visualization techniques in the last decade. With these models, the pathomechanistical function of some crucial bacterial virulence factors in VWF-mediated disease progression could be deciphered.

## Bacterial Binding to VWF Under Shear Flow

The release of VWF from endothelial WPBs is induced by host-derived hormones such as epinephrine and histamine and other plasma factors and is also triggered by pathogenic bacteria (32). For example, in 1991, Sporn et al. were the first to observe that the intracellular pathogen *Rickettsia rickettsii*, the main cause of the Rocky Mountain spotted fever, induces the release of VWF out of WPB of cultured endothelial cells [(33), Table 1]. Moreover, in our previous studies, we demonstrated that luminal VWF secretion from WPB of human lung endothelial cells is significantly increased in response to pneumococcal adherence and the cytotoxic effects of the pneumococcus toxin pneumolysin (45). These results strongly suggest that *in vivo*, the interaction between circulating bacteria in the bloodstream and the endothelial vasculature might directly lead to elevated VWF plasma levels.

**Table 1:** Bacterial VWF-binding proteins and function in adhesion and infectious diseases.

**Species**	**VWF binding factor**	**Function of VWF binding**	**References**
*Staphylococcus aureus*	SPA	Bacterial attachment to VWF strings in flow and collagen-rich subendothelium via catch bond mechanisms	(19, 34–36)
	VWbp	Flow-independent VWF binding of bacteria, coagulase activity, activation of host prothrombin, induction of fibrin formation, involved in pneumonia progression	(37–39)
*Coagulase-negative Staphylococci*	N.D.	Binding of soluble VWF to *S. epidermidis, S. haemolyticus*, and *S. hominis*	(40)
*Staphylococcus lugdunensis*	VWFbl	Attachment to endothelium under flow, adherence to cardiac valves and induction of endocarditis	(41, 42)
*Streptococcus pneumoniae*	enolase	Mediating adherence to endothelium and bacterial aggregation in blood	(20)
*Rickettsia rickettsii*	N.D.	Induction of VWF release	(33)
*Helicobacter pylori*	N.D.	Increase in VWF plasma levels, Induction of platelet aggregation	(43, 44)

*(N.D, not determined)*.

In this respect, the scientific question was raised whether the released VWF is directly subverted by the bacteria for their own benefit, i.e., as a binding site at the host endothelium, for platelet aggregation, or interference with the host coagulation. Indeed, Herrmann et al. were the first to demonstrate the binding of *S. aureus* bacteria to VWF coated surfaces and VWF in suspension (46). A short time later, a heparin-sensitive bacterial binding to soluble VWF was also reported for coagulase-negative *Staphylococcus* species, often associated with infections of prosthetic devices [(40), Table 1].

Bacterial adhesion to the vascular endothelium is of high importance for the pathology of blood-born infections, since this promotes bacterial settlement, induces inflammatory responses, and facilitates bacterial transmigration and dissemination into deeper tissue sites. It became obvious that blood-flow induced conformational changes of the VWF molecule, which are crucial for the physiological function of VWF in the bloodstream, might also be of high relevance for VWF-mediated bacterial adhesion. For a long time, it remained a technically challenging task to unreveal details of the bacterial interaction with the mechano-sensitive VWF under shear stress condition. But meanwhile, a variety of model systems have been established that enable the simulation of different physiological shear stress situations including sophisticated visualization techniques [for review, please refer to Bergmann and Steinert (47)]. The first experimental studies on the binding of multimerized VWF to platelets were performed with “Cone-and-Plate” viscometers in combination with flow cytometric quantifications (48). Viscometer-generated shear stress application was also combined with ristocetin-incubation of VWF. Ristocetin is an antibiotic produced by *Amycolatopsis lurida*, and is still used as the Gold standard in diagnostics of von Willebrand-disease (49). Ristocetin binds to VWF in a shear-stress-independent manner, thereby inducing the exposure of the VWF-mediated platelet binding site for thrombocyte recruitment and aggregation (49). Following the objective to quantitatively analyse the specific protein ligand-interaction with VWF under a defined medium flow, several surface-coating technologies have been established that create so-called “functionalized surfaces.” For example, Mascari and Ross have quantified the detachment of staphylococci from collagen in real–time using a parallel plate flow chamber combined with phase-contrast video–microscopy and digital image processing (50). The results provided evidence that staphylococci adhere directly to multimerized VWF strings and attach to collagen of the exposed subendothelium in blood-borne infections (34).

**Figure 1:**
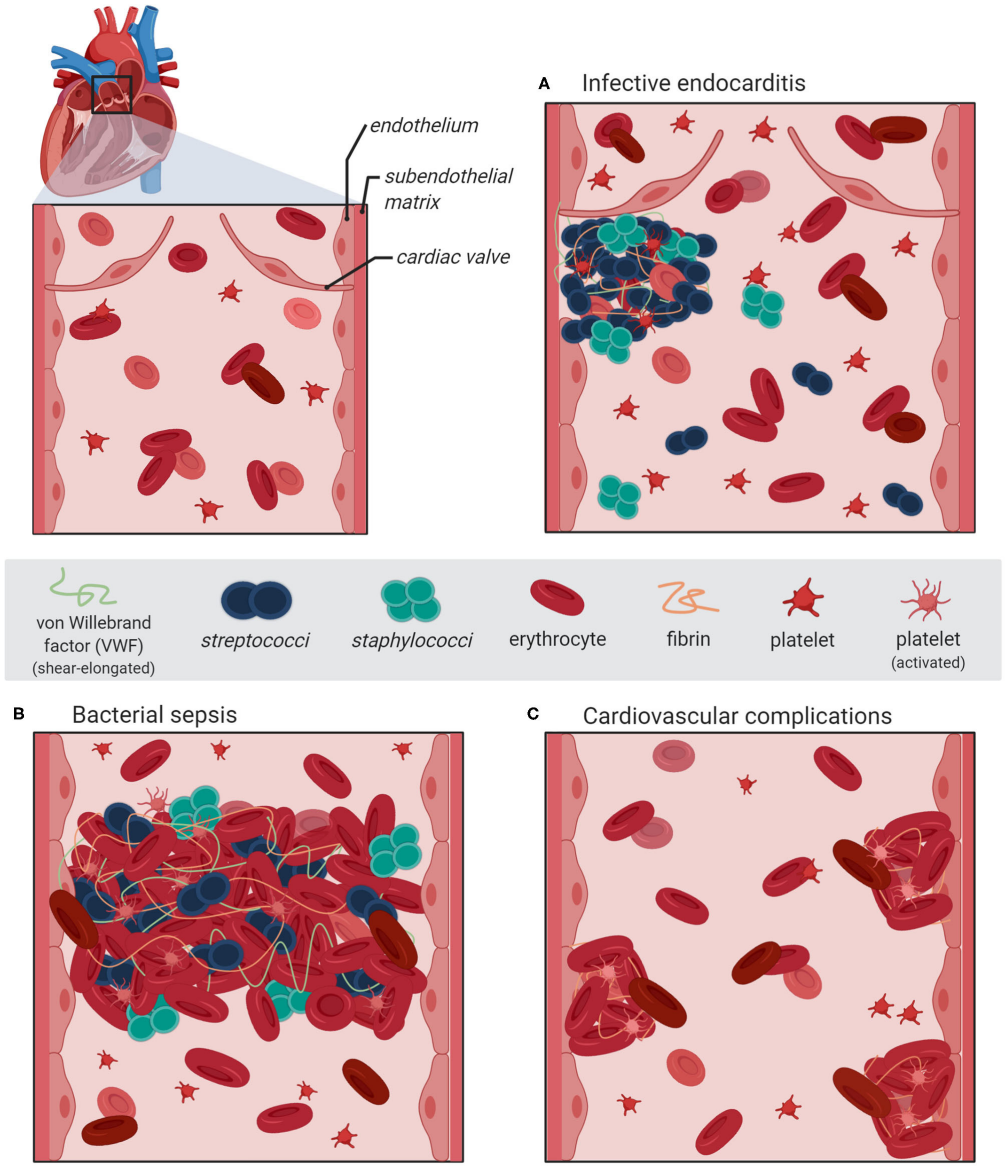
Schematic illustration of bacterial vascular infections and diseases associated with VWF-mediated adhesion of *S. aureus* and *S. pneumoniae* to activated endothelium. The section at the upper left represents a magnification of the heart pulmonary arerty with the artrioventricular valve. **(A)** An infective endocarditis is associated with VWF-mediated bacterial attachment to the activated valve endocard leading to the formation of inflammation-inducing bacterial vegetations containing platelets and fibrin. **(B)** During sepsis, bacterial adhesion to the inflamed vasculature is promoted via elongated VWF strings and induces the formation thrombi, which might lead to occlusions of the microvasculature. **(C)** Severe systemic bacterial infections are accompanied with recurring endothel activation leading to a longterm dysbalanced hemostasis, which increases the risk of cardiovascular complications such as major adverse cardiac effects. The figure was generated using bioRender Software.

In addition to the biochemical interaction studies, several *in vivo* mouse infection models employing *vwf* gene-deficient mice and platelet-depleted mice enable evaluation and monitoring of systemic consequences associated with hemostatic processes. The *in vivo* analyses revealed that *S. aureus* bacteria directly attach to cell-bound VWF of the endothelial vasculature (23, 51). Moreover, visualization of bacterial mouse infection via *intravital* microscopy confirmed that bacteria, which attached to VWF strings, resist shear stress-mediated clearance by the blood flow [(19), Figure 1]. Deeper insight into the pathophysiological consequences of the pneumococcus interaction with VWF was also provided by infection analyses using zebrafish larvae. *Danio rerio* serves as a suitable *in vivo* model, sharing high morphologic and functional similarity to the human endothelial tissue and both, intrinsic and extrinsic coagulation pathways (52–54). Microscopic real-time visualization of larval infection confirmed the recruitment of endothelial-derived VWF to circulating pneumococci and VWF-mediated attachment to the endothelial vessel walls (20).

## Bacterial VWF Binding Proteins and Binding Mechanisms

The bacterial interaction with components of the hemostasis *in vivo* augurs the presence of specific bacterial surface proteins, which mediate binding to VWF. The protein A (SPA) of *S. aureus* was identified as a bacterial VWF-binding protein. SPA elicits binding activities to both, the soluble and the surface-immobilized VWF [(35), Table 1]. Six years later, the VWF binding sites of protein A were narrowed down to the IgG-binding domain (55). Using single-molecule atomic force microscopy (AFM), Viela et al. further demonstrated that VWF binds very tightly to SPA via a force-sensitive catch bond mechanism, which involves force-induced structural changes in the SPA domains (36, 56). Meanwhile, protein similarities led to the assumption that several bacterial virulence factors may use this binding mechanism to resist clearance by high shear stress during infections (57).

In addition to SPA, a second staphylococcal VWF binding protein (VWbp) with coagulase activity was identified from a phage display-library screen in 2002 [(37, 38), Table 1]. Studies using functionalized surface-technology revealed that in contrast to SPA, VWbp appears to be of significant relevance for VWF recruitment rather than under static conditions (58). Likewise, pneumococci bind VWF under static conditions, and also recruit globular circulating VWF via the surface-exposed enolase [(20), Table 1]. This protein also mediates binding of pneumococci to plasminogen and to extracellular nucleic acids, which both promotes bacterial attachment to epithelial and endothelial cells (59). Moreover, similar to the staphylococcal VWbp, the VWF binding site for the pneumococcal enolase is located within the defolded A1 domain of VWF (19, 20, 60). All bacterial VWF-binding proteins identified so far are listed in Table 1.

In addition to the analyses of perfused VWF protein-coated, functionalized surfaces, the group of Schneider et al. established an air pressure-driven, unidirectional, and continuous pump system manufactured by the company ibidi® (19). In contrast to the formerly described flow systems that are employed to analyse protein-protein-interactions under shear stress conditions, the ibidi® pump technology enables sterile long term cultivation of VWF-producing endothelial cells, which can be incubated with bacteria and microscopically analyzed in real-time. As a result, this air-driven microfluidic pump device enabled the analyses of staphylococcal interaction with VWF on endothelial cell surfaces under shear stress conditions (19) and was also used to establish a pneumococcus cell culture infection model of primary endothelial cells in flow (20, 61). With this system, the attachment of pneumococci to multimerized VWF strings on the endothelial cell surface was successfully visualized and quantitatively evaluated. In accordance with the VWF binding characteristics of *S. aureus*, VWF binding to pneumococci is heparin-sensitive and depends on the amount of polysaccharide capsule expression (20). It is of note that pneumococcal attachment to VWF strings is also characterized by remarkable bond stability for longer time periods even at high shear flow parameters, which might be promoted by a concerted action of several additional, yet unidentified VWF-binding proteins (20). In addition, results of surface plasmon resonance binding studies and cell culture infections studies in flow revealed that the pneumococcus enolase interacts with both, globular circulating VWF and with VWF strings with comparable avidity. Based on the observation that multi adhesive proteins such as the bacterial enolase are already detected on the surface of various bacterial species, it can be assumed that the bacterial interaction with VWF is part of a general mechanism with pivotal relevance for pathophysiology.

## Effect of Staphylococcal and Streptococcal Interaction With VWF on Coagulation and Vascular Diseases

As summarized in Table 1, VWF binding to bacteria has only been studied to detail for staphylococci and streptococci. Taking clinical symptoms into account, different functional aspects of the bacterial interaction with VWF can be directly or indirectly correlated with at least three severe infection diseases: infective endocarditis, bacterial sepsis, and cardiovascular complications.

Infective endocarditis is regarded as a paradigm of bacterial diseases associated with vascular inflammation and VWF-interaction (24). Most of the acute infective endocarditis are caused by *S. aureus* and are associated with up to 100% mortality rate if left untreated (21, 22). Compared to that, infective endocarditis caused by *S. pneumoniae* is rare but no less severe (27, 28). Infection of the heart valves is initiated by the attachment of circulating bacteria to the endocardium and the formation of bacterial vegetations, which are embedded in fibrin and platelets (Figure 1A). During disease progress, the vegetations induce further inflammatory processes, which result in ulceration, rupture, and necrosis of the valve cusps (62, 63). Experimental shear stress determination using native porcine aortic valve models revealed that even in a healthy human vasculature, the systolic shear stress at the heart valve leaflet can reach up to 21.3 dyn/cm^2^ at the aortic site and up to 92 dyn/cm^2^ at the ventricular site (64, 65). Similar to the activation of specific proinflammatory and procoagulant protein expression patterns of endothelial cells, the hemodynamic forces also promote the activation of endocardial Notch-dependent signaling pathways in the endocardial cells of the atrio-ventricular valve (66). The observed magnitude of shear stress is sensed by the mechano-responsive VWF and induces stretching and multimerization of VWF proteins. Thereby, VWF displays crucial binding sites for bacterial surface adhesins and mediates bacterial attachment to the heart valve. In line with this, visualization of staphylococcal mouse infection via 3D confocal microscopy confirmed the adhesion of fluorescent *S. aureus* to murine aortic valves (23). The mouse infection studies further demonstrated that following valve damage, VWF and fibrin are both deposited on the damaged valve endocardium and serve as attachment sites for *S. aureus* [(23, 51), Figure 1A]. Moreover, endothelial cell culture infections and *intravital* microscopy of bacterial mouse infection confirmed that staphylococci and pneumococci resist shear stress-mediated clearance by the blood flow by binding to VWF strings at the endothelial vessel walls (19, 20, 51). Following disease progress, the VWF-mediated bacterial attachment also promotes the recruitment of large amounts of platelets, capturing *S. aureus* to the valve surface [(23, 24, 67, 68), Figures 1A,B]. The observation that among the staphylococci, only *S. aureus* and *S. lugdunensis* are able to bind VWF might, in part, explain why these bacteria are more effective in causing endocarditis than other staphylococci (41).

Bacterial VWF binding is also involved in the formation of large platelet aggregates within the blood circulation. In this respect, the formation of bacterial-induced platelet aggregates and the depletion of clotting factors from blood represents a crucial pathomechanism, which is directly attributed to disease symptoms typical for bacterial sepsis. For example, staphylococcal sepsis is associated with an increase in coagulation activity and an enhanced thrombosis (Figure 1B; Table 1). It is assumed that the *Staphylococcus*-induced dysregulated activation of systemic thrombosis leads to thrombotic microangiopathy, which is associated with an accelerated fibrinolysis and bleeding tendency, referred to as disseminated intravascular coagulation [DIC, (69)]. Moreover, this bacterial mechanism is also assumed to directly induce the formation of abscesses [(39, 70–73), Figure 1B]. A similar formation of blood clots, reaching up to 10 μm in diameter, was observed in pneumococcus infection of *Danio rerio* larvae (20). Based on these data, we suppose that the VWF-mediated bacterial aggregate formation in the blood circulation of the zebrafish cause a partial or complete occlusion of the larval microvasculature. Thus, in severe cases of staphylococcal and pneumococcal septicaemiae, the vascular occlusion of small blood vessels throughout the body represents a life-threatening disease symptom, which might lead to multi-organ failure, resulting in high mortality rates of up to 50% [(74–77), Figure 1B]. Bacterial aggregate formation in sepsis and infective endocarditis, in particular, are also prime examples of the strong connection between the hemostatic system and innate immunity, which is referred to as immune thrombosis (78). It is coincidently proposed that the infection-induced coagulase activity mediates bacterial capture within a fibrin meshwork, which enables this pathogen to disseminate via thromboembolic lesions and to resist opsonophagocytic clearance by host immune cells (73). On the other hand, platelets are the crucial mediators of the innate defense against staphylococci by releasing microbicidal proteins from alpha granules that kill the bacteria (79). On the first view, it appears to be contradictory that bacteria induce a clotting mechanism, which is originally developed as an anti-bacterial immune defense mechanism of the host. However, the biochemical and physiological attributes of the fibrin meshwork formed by staphylocoagulases are thought to be distinct and less solid than those generated by thrombin (80). Therefore, instead of containing the infection, immune thrombosis might rather create the optimal environment for bacteria to survive and to evade the immune defense of the host (24).

It is supposed that the bacterial infection mechanism leading to vascular dysfunction and enhanced activation of inflammation might also be implicated in developing cardiovascular complications (Figure 1C). An increasing number of clinical studies solidify the observation that pneumococci induce vascular inflammation of the endothelial vessel wall, including the aorta (81), and that severe pneumococcal infections such as pneumoniae and septicemiae lead to a higher risk for major adverse cardiac effects (MACE) such as myocardial infarction, ischemic stroke, and arterial thrombosis (29–31).

Since elevated VWF plasma levels are known to be associated with an increased risk for MACE (15), the endothelial VWF release induced by pneumococcal attachment and by pneumolysin activity might be partially responsible for the pathologic effects on the cardio vasculature (45). As further explanation, functional variants of VWF have been identified, which elicit differences in the protein conformation and shear sensitivity. These variants are associated with increased platelet aggregate size and the occurrence of these VWF variants correlates with a higher risk of thromboembolisms including myocardial infarction and stroke (82). In line with these observations, it can be assumed that bacterial interaction with VWF might effect the hemostatic function in various ways, i.e., by sterical hindrance of the platelet binding site, by alteration of the VWF conformation, and by inhibition of dimerization and multimerization activities, thereby increasing the risk for cardiovascular complications.

## Conclusions

VWF is a live-saving key component of coagulation and immune thrombosis in response to vascular injury and inflammation. Bacterial interaction with VWF is of high medical and scientific importance since this interaction is directly associated with specific clinical manifestations and long-term complications of infectious diseases. It has been demonstrated that binding of S. aureus and *S. pneumoniae* to VWF strings is controlled by hydrodynamic flow conditions. So far, at least three bacterial pathomechanisms involving host-derived VWF can be named: (i) binding to multimerized VWF strings mediates bacterial attachment to endothelial surfaces in blood flow–a major prerequisite of bacterial colonization, inflammation, and dissemination. (ii) VWF recruitment facilitates bacterial capture within clotted blood, thereby preventing bacterial clearance via immunothrombosis; (iii) recruitment of intravascular VWF induces bacterial aggregate formation, which leads to occlusion of microcapillaries and impaired blood supply. Although several sophisticated technologies such as microfluidic systems and binding force determinations already provided most valuable insights into the cell biological and biochemical details, the multifactorial complexity of the bacterial interaction with VWF still remains a challenging subject of ongoing scientific research.

## Author Contributions

SB and MS contributed to text conception and wrote the text. IR has generated the figure and critically revised the text. All authors contributed to manuscript revision, read, and approved the submitted version.

## Conflict of Interest

The authors declare that the research was conducted in the absence of any commercial or financial relationships that could be construed as a potential conflict of interest.
